# Recent Progresses in NIR-I/II Fluorescence Imaging for Surgical Navigation

**DOI:** 10.3389/fbioe.2021.768698

**Published:** 2021-11-01

**Authors:** Songjiao Li, Dan Cheng, Longwei He, Lin Yuan

**Affiliations:** ^1^ Cancer Research Institute, Department of Pharmacy and Pharmacology, The First Affiliated Hospital, Hunan Province Cooperative Innovation Center for Molecular Target New Drug Study, Hengyang Medical School, University of South China, Hengyang, China; ^2^ The Affiliated Nanhua Hospital, Hengyang Medical School, University of South China, Hengyang, China; ^3^ State Key Laboratory of Chemo/Biosensing and Chemometrics, College of Chemistry and Chemical Engineering, Hunan University, Changsha, China

**Keywords:** surgical navigation, NIR-I, NIR-II, tumor, fluorescence imaging, image-guided cancer surgeries

## Abstract

Cancer is still one of the main causes of morbidity and death rate around the world, although diagnostic and therapeutic technologies are used to advance human disease treatment. Currently, surgical resection of solid tumors is the most effective and a prior remedial measure to treat cancer. Although medical treatment, technology, and science have advanced significantly, it is challenging to completely treat this lethal disease. Near-infrared (NIR) fluorescence, including the first near-infrared region (NIR-I, 650–900 nm) and the second near-infrared region (NIR-II, 1,000–1,700 nm), plays an important role in image-guided cancer surgeries due to its inherent advantages, such as great tissue penetration, minimal tissue absorption and emission light scattering, and low autofluorescence. By virtue of its high precision in identifying tumor tissue margins, there are growing number of NIR fluorescence-guided surgeries for various living animal models as well as patients in clinical therapy. Herein, this review introduces the basic construction and operation principles of fluorescence molecular imaging technology, and the representative application of NIR-I/II image-guided surgery in biomedical research studies are summarized. Ultimately, we discuss the present challenges and future perspectives in the field of fluorescence imaging for surgical navigation and also put forward our opinions on how to improve the efficiency of the surgical treatment.

## Introduction

As an extremely aggressive disease for human health, cancer is still one of the most fatal diseases and the main cause of death across the world (Torre et al., 2015; Torre et al., 2016; de Groot et al., 2018; Feng et al., 2019; Rawla, 2019). On the basis of the recent investigation conducted by the World Health Organization (WHO), there was an increase of about 18 million cases of cancer globally, including almost 10 million patients dying from cancer in 2018 (Bray et al., 2018; Curry et al., 2019). Although some tumor surgeries have been advanced methodologically in the past few decades, there is no significant overall growth in the cure (Esserman et al., 2013; Kamzan and Ng, 2021). As a dynamic process, cancer progression experiences genetic mutation or excision, elementary tumor generation, vascularization, aggression, and secondary tumor generation (Hanahan and Weinberg, 2011; Faubert et al., 2020). Thus, through precise distinction of tumor areas and complete surgical resection of tumor tissues with negative margins, the prognosis of the disease could be improved while decreasing the rate of recurrence.

For satisfying the abovementioned requirements, tumor diagnosis and therapeutic evaluation have been developed and employed in different medical imaging techniques, such as magnetic resonance imaging (MRI), computed tomography (CT), positron emission tomography (PET), ultrasonic imaging (USI), and single photon emission computed tomography (SPECT) (Owens et al., 2016; Feher and Sinusas, 2017; Yun and Kwok, 2017; He et al., 2018; Xia et al., 2019). Preoperative diagnosis can apply those imaging approaches, whereas fluorescence image-guided surgery helps surgeons to accurately identify the cancerous tissue by using fluorescence approaches for intraoperative imaging (Nagaya et al., 2017; Hu et al., 2018; Mangeolle et al., 2018; Wang C. et al., 2019; Hernot et al., 2019; Olson et al., 2019). Excitation/emission wavelengths within the NIR-I or NIR-II scopes have been adopted to develop a lot of fluorescence image-guided surgery dye platforms, including small-molecule fluorogenic chromophores (such as indocyanine green (ICG), cyanine 7 (Cy7), hemicyanine, dicyanomethylene pyran (DCM), and phenothiazine (PTZ) derivatives) and fluorogenic nanomaterial (such as aggregation-induced emission (AIE) dots, amphipathic polymer, lanthanide nanoparticle, Ag_2_S nanochain, and DNA-functionalized nanoparticle). Small-molecule fluorogenic probes usually perform quick feedback, have high sensitivity and excellent selectivity, and easy adjustment of optical properties through structural modification; however, their response emission and imaging applications might suffer the intrinsic disadvantages of organic compounds, such as poor water solubility generated by large π-conjugated structures, low quantum yield generated by non-rigid structures, fake signal induced by unstable chemical structures and environment-vulnerable emission, self-quenching induced by small stokes shift, and so on. In comparison, the NIR nanoprobes based on fluorogenic nanomaterials or small-molecule chromophores modified by nanoparticles tend to own high stability, good water dispersibility, good biocompatibility, and high fluorescence brightness, which leads to the capability of long-term *in vivo* imaging. Meanwhile, there are some disadvantages to the fluorescent nanoparticles, for example, it takes long time to penetrate the cell member for the large-size particles, and nanoparticles are metabolized slowly *in vivo* and substantial cumulation in the body resulting in organ injury.

**SCHEME 1:**
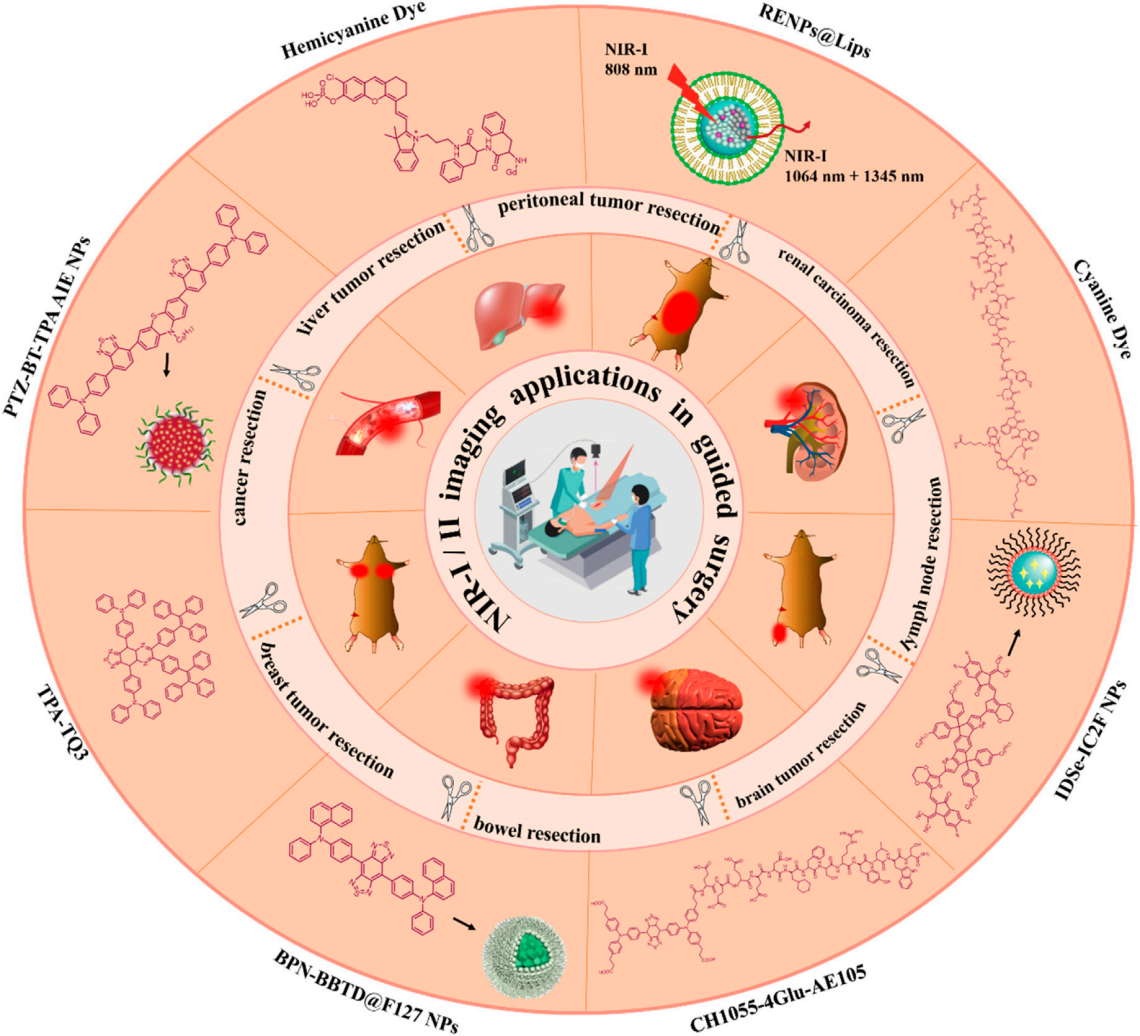
Illustrative representation of NIR-I/II fluorescence imaging technology and its applications in guided surgery.

Fluorescence imaging in the first near-infrared region (NIR-I, 650–900 nm) has received widespread attention in biomedical research studies because of its quick feedback, high sensitivity, harmless radiation, and lower cost (Liu et al., 2019; Ji et al., 2020; Jackson et al., 2021; Yan et al., 2021; Yu et al., 2021; Zeng et al., 2021). For example, NIR-I fluorophores utilizing reasonable design strategies were extensively used for biomedical usage including precise real-time sentinel lymph nodes/tumor description and intraoperative image-guided surgical resection of sentinel lymph nodes/tumor tissues (Li et al., 2021). Relatively deep penetration and high imaging quality can be achieved with fluorophores with emissions in the NIR-I region by comparing with visible wavelengths. In addition, recent research studies indicate that high-quality fluorescence images and a signal-to-background ratio (SBR) can be induced by fluorescence imaging at the second near-infrared region (NIR-II, 1,000–1700 nm) than at the NIR-I region (Huang and Pu, 2020; Qian et al., 2020; Zhang et al., 2020). The applications of this innovative NIR-II region in biomedical imaging have significantly improved the temporal and spatial resolution and penetration depth because of negligible tissue absorption, weakened scattering, and minimum autofluorescence (Ding et al., 2018b; Wan et al., 2019; Dai et al., 2021). As a rapidly developing technology, near-infrared fluorescence-guided surgery helps surgeons in determining the tumor margins of some cancer types and the lesions of other common diseases with great accuracy (Scheme 1) (Li et al., 2019; Zhu et al., 2019; Wu and Zhang, 2020; Xu et al., 2020; Yang et al., 2021).

Herein, this review summed up the recent progress in developing fluorescence imaging-guided surgery by two kinds of NIR imaging (NIR-I and NIR-II fluorescence). We will briefly cover background and design considerations focusing on representative examples applied in fluorescence image-guided surgery. The performance comparison of fluorescent probes used in related highlight researches is summarized in Table 1. Finally, the discussion on the current challenges and future perspectives in the field of fluorescence imaging-guided surgical navigation is showed in the final section, and the opinions on how to improve the efficiency of the surgical treatment are also put forward by us.

**TABLE 1:** Summary performance comparison of fluorescence image-guided surgery dye platforms.

Probe name	Ex/Em (nm/nm)	Imaging modality	Analytes	Surgical application	References
BL-760	760/783	FL	None	Hepatobiliary surgery	Luciano et al. (2019)
TER-SA	760/780	FL	α_v_β_3_ integrin	Renal carcinoma resection	An et al. (2020)
TPA-TQ3 NPs	600/800	FL/PA	none	Breast tumor resection	Gao et al. (2021)
VGT-309	750/773	FL	Cathepsins	Breast tumor resection	Suurs et al. (2020)
P-Cy-FF-Gd	688/710	FL/MR	Alkaline phosphatase	Liver tumor resection	Yan et al. (2019)
BH-NO2@BSA	680/791	FL/PA	Nitroreductase	Liver tumor resection	Zeng et al. (2020)
YH-APN	445/650	FL	Aminopeptidase N	Liver tumor resection	Li et al. (2020)
IRDye800CW-SAHA	760/801	FL	Histone deacetylase	Liver tumor resection	Tang et al. (2020)
TPE-Ph-DCM	465/671	FL	None	Peritoneal tumor resection	Ni et al. (2019)
DSPE-PEG-AIE dots	445/650	FL/BL	None	Peritoneal tumor resection	Chen et al. (2020)
PTZ-BT-TPA	480/650	FL/BL	None	Peritoneal tumor resection	Qi et al. (2020)
OTPA-TQ3 NPs	650/900	FL/RL	None	Osteosarcoma resection	Qi et al. (2019)
H3-PEG2k	760/1,023	FL	None	Mammary carcinoma resection	Zeng et al. (2019)
BPN-BBTD@F127	710/930	FL	None	Bowel resection	Fan et al. (2021b)
ICG	808/980	FL	None	Liver tumor resection	Hu et al. (2020)
CH1055-4Glu-AE105	750/1,055	FL	None	Glioblastoma resection	Kurbegovic et al. (2018)
DCNPs	808/1,060	FL	None	Lymph node resection	Wang et al. (2018)
IDSe-IC2F NPs	793/1,010	FL	None	Lymph node resection	Fan et al. (2021a)
PDFT1032	808/1,032	FL	None	Osteosarcoma resection	Shou et al. (2018)
RENPs@Lips	808/1,064	FL	None	Osteosarcoma resection	Li et al. (2019)
RBCp	980/835	FL	None	Osteosarcoma resection	Wang et al. (2019b)
SCH4	739/1,050	FL	None	Osteosarcoma resection	Ding et al. (2018a)
^68^Ga-CHS2	808/1,055	FL/PET	None	Osteosarcoma resection	Sun et al. (2018)
IR-BEMC6P	808/1,025	FL	None	Osteosarcoma resection	Liu et al. (2021)
2TT-m, oC6B NPs	777/1,059	FL	None	Peritoneal tumor resection	Liu et al. (2021)
pNIR4-PAE NPs	750/1,040	FL	None	Peritoneal tumor resection	Wen et al. (2019)
APP-Ag_2_S-RGD	808/1,200	FL	None	Peritoneal tumor resection	Ling et al. (2020)
FEAD1	1,094/1,200	FL	None	Peritoneal tumor resection	Ling et al. (2020)

## NIR-I Fluorescence Imaging Technology for Guided Surgery

There is a wide application prospect of fluorescence imaging technologies in the biomedical field for the monitoring of physiological and pathological courses at the molecular and cellular levels by right of its intrinsic merits, including high spatiotemporal resolution, favorable sensitivity, and biocompatibility. Particularly, NIR-I emission (650–900 nm) instead of UV or visible fluorescence molecules is interested in applying *in vivo* fluorescence imaging because of its ability to greatly eliminate autofluorescence, decrease photon scattering, and enhance in-depth tissue penetration (Liu et al., 2019; Ji et al., 2020; Jackson et al., 2021; Yan et al., 2021; Zeng et al., 2021). As stated earlier, *in vivo* fluorescence imaging utilizing NIR light with longer wavelengths (650–900 nm), rather than ultraviolet or visible light, is effective in minimizing interference from biological optical absorbers. The NIR-I fluorescent probe is able to analyze the selective visualization of the tumor location and metastatic foci satisfactorily, which has attracted widespread attention for image-guided surgeries. This section focuses on the current representative examples of NIR-I fluorescence imaging technology on surgical navigation.

### Hepatobiliary Surgery

**FIGURE 1:**
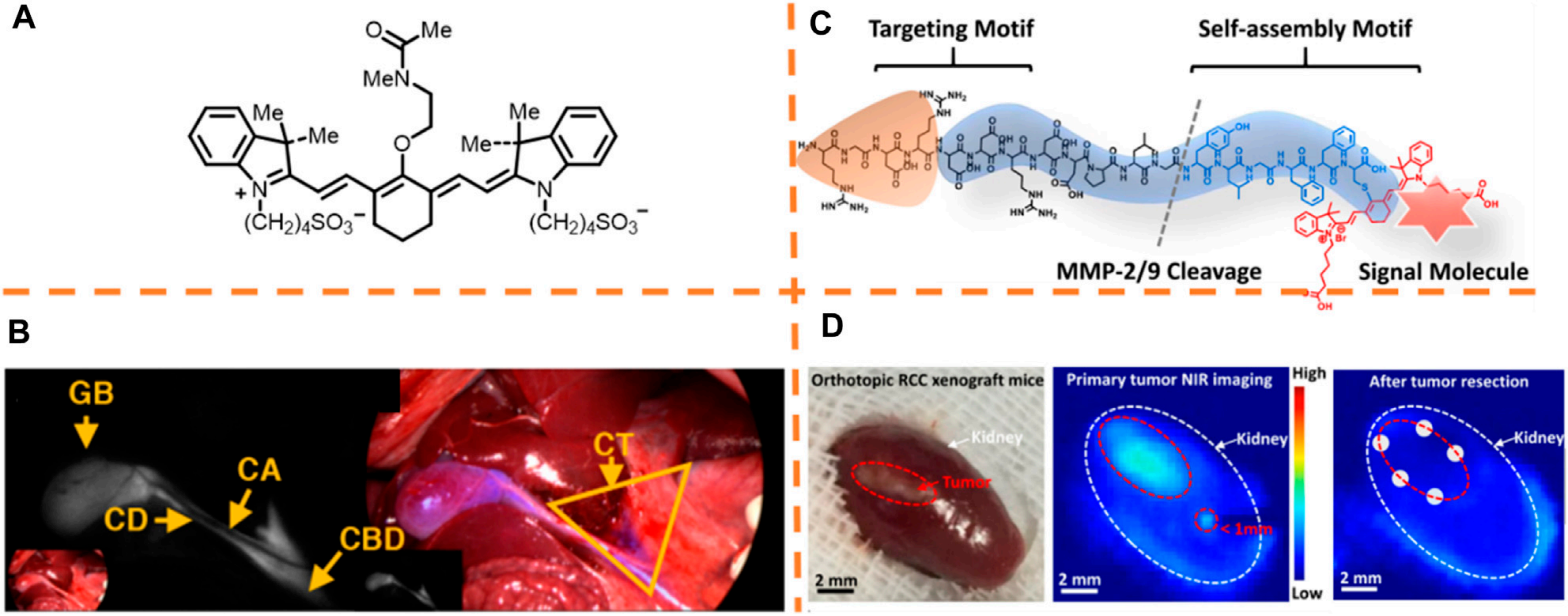
**(A)** Chemical structures of the fluorescent dye BL (bile label)-760. **(B)** Dissection on the liver hilum (left) and liver resection surgery (right). Adapted with permission from Luciano et al. (2019). **(C)** Schematic illustration of the molecular design. **(D)** Typical macroscopic figures of the orthotopic 786-O xenograft mice sample (left). Related NIR fluorescence figures of the orthotopic 786-O xenograft mice sample after intravenously governing the TER measure (middle). Arbitrary biopsy (*n* = 5) at the margin (right). Adapted with permissions from An et al. (2020).

Cha et al. reported a biliary tract–targeting NIR fluorescent chromophore BL (bile label)-760 (λ_*ex*_/λ_*em*_, 760 nm/783 nm) (Figure 1A) (Luciano et al., 2019). Different from ICG, BL-760 is superior with regard to its capacities, such as lower function doses (90 μg/kg), much less duration to biliary excretion, and a higher target-to-background ratio (TBR, 4.48 at 10 min) of the cystic duct relative to the liver parenchyma. For demonstrating its efficacy in hepatectomy, authors conducted the hepatic resection surgery of the left lobectomy in pigs. During the surgery, the intrahepatic ducts were clearly highlighted, and bile leakage was easily detected. After carefully dissecting the liver hilum, Calot’s triangle was exposed and visualized successfully (Figure 1B). All results validated that BL-760 has highly promising properties for intraoperative navigation during hepatobiliary surgery.

### Renal Carcinoma Resection

To develop fluorescent molecular tools for image-guided surgeries, Wang et al. reported an NIR probe for imaging renal cell carcinoma based on peptides (λ_*ex*_/λ_*em*_, 760 nm/780 nm) (Figure 1C) (An et al., 2020). On the basis of the design given by the authors, the peptide specifically binds to integrins overexpressed on the cancer cells, and the peptide required to release the self-assembled peptide enclosed with the cyanine dye was cleaved by MMP2/9 enzyme overexpressed cancer cells to generate fluorescent nanoparticles on the surface of the cells. After determining the *in-situ* enzyme-caused self-assembly of the NIR peptide probes on cancer cells, the probes on tumor lesions in a murine model were tested by the authors. Whether the NIR peptide probe could detect tumor precisely in orthotopic RCC models was further investigated by the authors. By virtue of the NIR imaging system, the clear visualization of the whole tumor margins could be performed, and a satellite lesion (<1 mm) could be detected (Figure 1D). The probe, playing an excretion-reducing role in the kidney, enabled the identification of little lesions for complete tumor resection and greatly decreased the postoperative recurrence of the tumor by comparison with conventional surgeries. Besides, the tumor-specific excretion-retarded (TER) role was verified by applying a model of *ex vivo* kidney perfusion.

### Breast Tumor Resection

**FIGURE 2:**
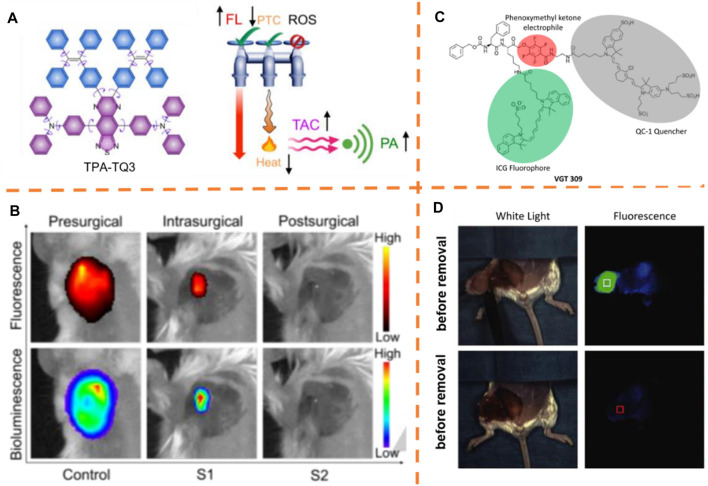
**(A)** Chemical structure of TPA-TQ3. **(B)** Typical pre-, intra- and post-surgical fluorescence/bioluminescence images of orthotopic 4T1 breast tumor. Adapted with content from Gao et al. (2021). **(C)** Chemical structure of VGT-309. **(D)** White light and fluorescent images of the surgical area before and after tumor removal. Adapted with permission from Suurs et al. (2020).

Ding et al. reported a superb probe (TPA-TQ3 NPs, λ_*ex*_/λ_*em*_, 600 nm/800 nm) employing a donor–acceptor (D-A) construction strategy in which triphenylamine (TPA) and thiadiazoloquinoxaline (TQ) were selected as the donor and acceptor moieties, respectively (Figure 2A) (Gao et al., 2021). The mixture with heavy intramolecular action showed an enlarged photoacoustic (PA) signal by enhancing the thermal-to-acoustic shift, and the fluorescence emission intensity also was enhanced because of an aggregation-caused emission (AIE) signature. With high sensitivity and equipment flexibility of fluorescence imaging, it becomes an optimal choice for quick intraoperation imaging, which has also been applied clinically. As shown in Figure 2B, tiny tumor tissues were detected unambiguously in real-time due to intraoperative NIR fluorescence imaging. It was observed that the fluorescence signal was on a good basis of the bioluminescence from the tumor cells expressed by luciferase, which determined that the fluorescence-marked regions were indeed tumor tissues. With the synchronously large PA signal and fluorescence brightness of TPA-TQ3 NPs, image-guided surgery was precisely carried out. This research study emphasizes a novel design guide of intramolecular motion, amplifying the PA role.

In another study, Dam et al. formulated a cathepsin-targeting fluorescent cysteine cathepsin probe, VGT-309 (λ_*ex*_/λ_*em*_, 750 nm/773 nm) (Figure 2C) (Suurs et al., 2020). The authors investigated the potential applications of cathepsin-directed imaging with VGT-309 for surgically resecting the 4T1 tumor. Figure 2D shows the image-guided surgeries of mice after administering the probe injection. The fluorescent signal well-delineated the tumor before removal. After tumor removal, the fluorescent signal in the tumor bed declined greatly. Cathepsins are overexpressed in breast cancer, and this outcome supports the potential practical applied value of VGT-309 for fluorescence imaging-guided surgeries among patients suffering from breast cancer.

### Liver Tumor Resection

**FIGURE 3:**
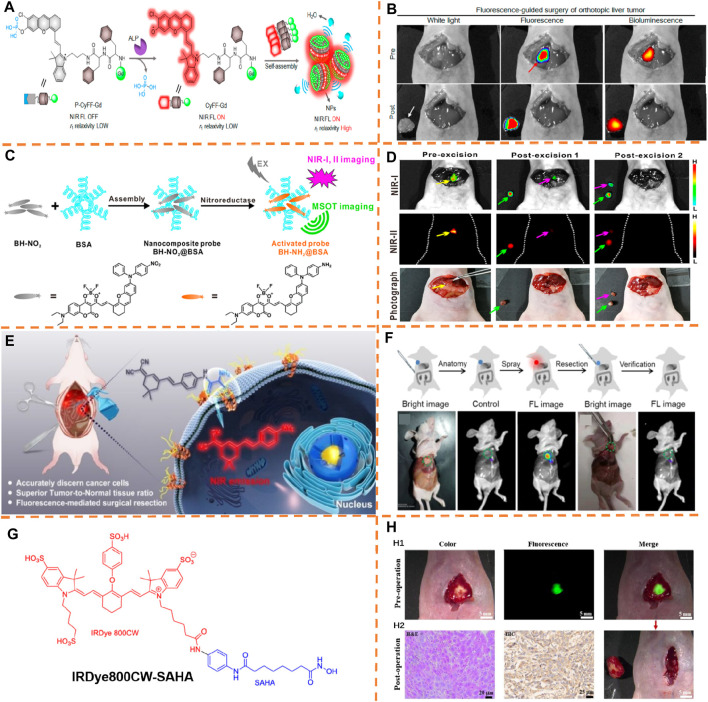
**(A)** Chemical construction of P-CyFF-Gd and suggested alkaline phosphatase–adjusted fluorogenic reaction and *in situ* self-gathering of P-CyFF-Gd into NPs. **(B)** Fluorescence-guided surgery of the orthotopic liver tumor. Adapted with consent from Yan et al. (2019). **(C)** Schematic explanation of production of the nanocomposite probe BH-NO_2_@BSA and its response to nitroreductase. **(D)** Image-guided tumor resection surgery using the nanocomposite probe. Adapted with consent from Zeng et al. (2020). (E) Schematic explanation indicating the course of activating YH-APN for APN among cancer cells. **(F)** *In vivo* imaging of mice bearing tumor and image-guided surgeries realized by the spray of YH-APN. Adapted with consent from Li et al. (2020). **(G)** Chemical structure of IRDye800CW-SAHA. **(H)** H1: Intraoperative NIR fluorescence imaging of the liver tumor after injecting IRDye800CW-SAHA. H2: immunohistochemical staining for histone deacetylase 6 and H&E staining among tumor tissues (Tang et al., 2020).

Ye et al. developed an NIR fluorescence/MRI dual-modal probe for *in vivo* fluorescence imaging (Figure 3A) (Yan et al., 2019). The small-molecule probe (P-Cy-FF-Gd) includes an alkaline phosphatase activatable NIR fluorophore (λ_*ex*_/λ_*em*_, 688 nm/710 nm), a self-gathering dipeptide, and a magnetic resonance (MR) contrasting agency. The authors declared that the overexpression of endogenous ALP on cancer cell membranes and the elimination of the phosphate group on the fluorophore (Cy) could lead to the self-assembly of the dephosphorylated probe (CyFF-Gd) into nanoparticles, resulting in simultaneous fluorescence enhancement >70-fold and about 2.3-fold r1 increase in relaxivity. P-CyFF-Gd was employed for mapping orthotopic liver tumor margins in intraoperative mice and dissecting the orthotopic HepG2/Luc tumor in intraoperative mice under the guidance of fluorescence imaging by the direct spraying of P-CyFF-Gd on the liver. Thirty minutes later, strong fluorescence, precisely delineating tumor margins in the liver, was produced and could effectively and surgically resect the tumor tissue (Figure 3B).

It is important to exert more focus on digging the approaches with the precise image of the tumor, which is crucial for resecting and treating the tumor. An activatable fluorescent nanoprobe BH-NO_2_@BSA (λ_*ex*_/λ_*em*_, 680 nm/791 nm) including bovine serum albumin (BSA) and a molecular probe (BH-NO_2_) was synthesized by Wu et al., which could particularly answer nitroreductase (NTR) in liver tumor cells and generate stressed NIR and multispectral optoacoustic tomography (MSOT) signals (Figure 3C) (Zeng et al., 2020). Applying this nanoprobe, the locations of orthotopic liver tumors were accurately determined by 3D MSOT images at the preoperative stage, and after that the tumors were completely removed under the intraoperative guidance of NIR-I and -II bioimaging (Figure 3D).

Most recently, Peng et al. disclosed a DCM-based fluorescent activatable probe by aminopeptidase N (APN) for image-guided surgeries through *in situ* spraying (Li et al., 2020). Guided by the fluorescence signal after probe activation, the probe YH-APN (λ_*ex*_/λ_*em*_, 445 nm/650 nm) (Figure 3E) was sprayed to precisely eliminate the tumor. YH-APN is capable of specially lighting up the *in situ* solid tumor in real-time with ratio values of 13.86, 4.42, 6.25, and 4.99 for subcutaneous transplantation tumor, hepatic metastasis, and splenic metastasis to normal tissue, respectively, *via in situ* spraying techniques (Figure 3F). More importantly, YH-APN is able to trail metastasis tumor tissues with a diameter of less than one mm, and the fluorescent tissues from the mouse could be precisely removed with a scalpel, explaining the potential of YH-APN to be used as a fluorescent contrast agent in surgical resection. The application of enzyme-activatable fluorescent probes might be promoted by such research studies to diagnose the tumor and surgeries under the guidance of images.

Tian et al. developed a new histone deacetylase–directed near-infrared probe IRDye800CW-SAHA (λ_*ex*_/λ_*em*_, 760 nm/801 nm) for hepatocellular carcinoma imaging and fluorescence image-guided surgery (Figure 3G) (Tang et al., 2020). Encouraged by these *in vivo* imaging outcomes, the authors induced the intraoperative liver tumor imaging and image-guided resection with IRDye800CW-SAHA. As displayed in Figure 3H1, great comparison between the tumor region and normal ambient tissue after the administration of the probe was showed by the intraoperative NIR fluorescence imaging, and the clear visualization and ready distinction of the liver tumor from the nearby normal tissues were drawn. After surgical resection of the fluorescence-positive tumor tissues, the tumor was kept ahead for the histologic (H&E staining) exploration (Figure 3H2), which determined that the resected tissues were tumor-directed tissues, indicating the smooth execution of hepatocellular carcinoma resection by fluorescence navigation. The authors validated HDAC6 expression in hepatocellular carcinoma tumor tissues by performing immunohistochemical analysis, which exerted a significant effect on hepatocellular carcinoma pathogenesis and progression.

### Peritoneal Tumor Resection

**FIGURE 4:**
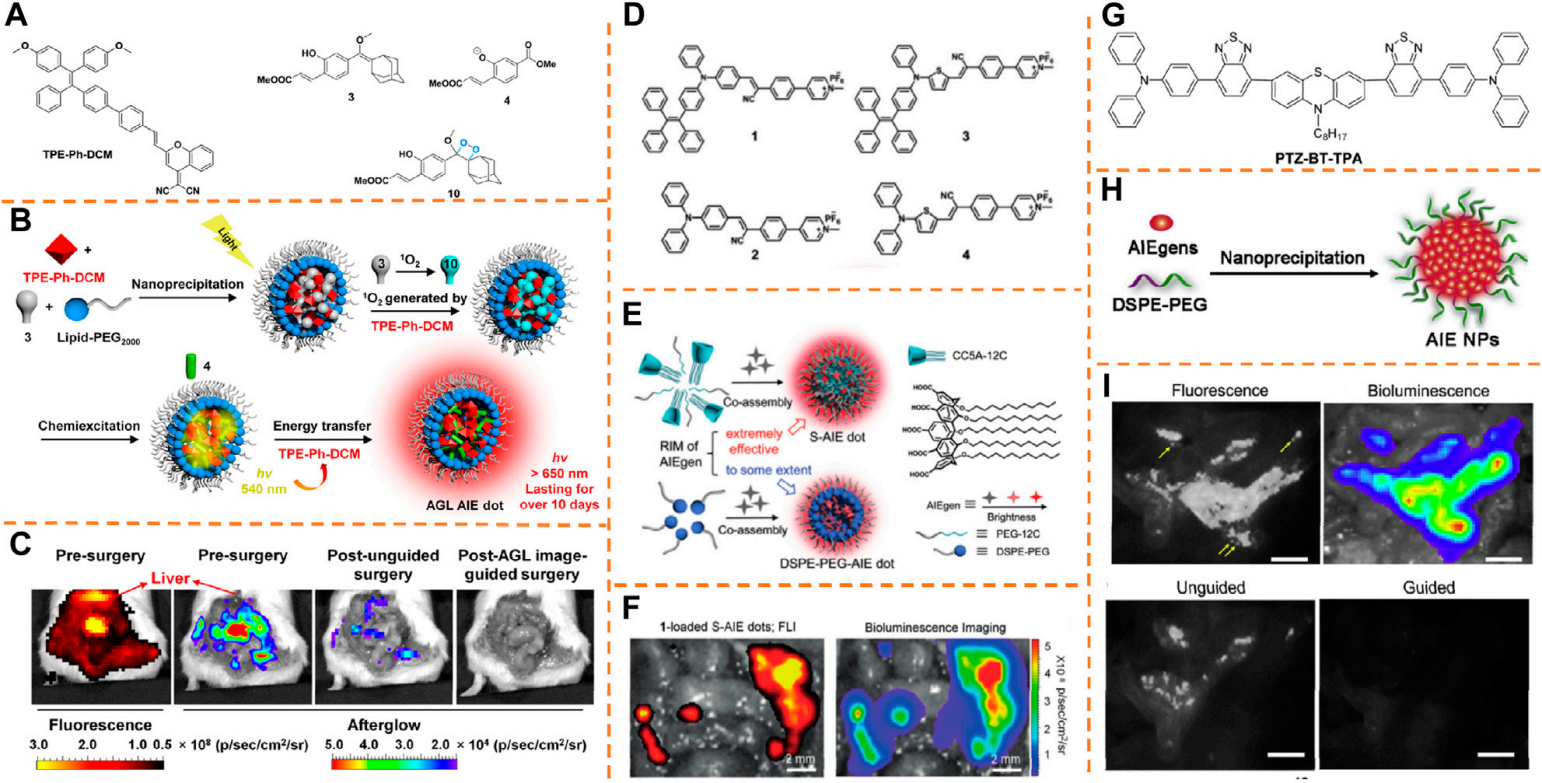
**(A)** Chemical structures of compounds 3, 4, 10, and TPE-Ph-DCM. **(B)** Schematic explanation of the mechanism for enlarged NIR afterglow luminescence of the AGL AIE dots. **(C)** Representative NIR fluorescence and afterglow imaging of the peritoneal carcinomatosis–bearing mice. Adapted with consent from Ni et al. (2019). **(D)** Chemical constructions of the AIEgens. **(E)** S-AIE dots and DSPE-PEG-AIE dots and chemical construction of CC5A-12C. **(F)** Fluorescence imaging and bioluminescence imaging of the intraperitoneal tumor nodules from mice after intravenously injecting 1-loaded S-AIE dots for 24 h. Adapted with consent from Chen et al. (2020). **(G)** Chemical structure of PTZ-BT-TPA. **(H)** Schematic illustration preparing AIE NPs with a nanoprecipitation approach. **(I)** Representative fluorescence and bioluminescence imaging of the peritoneal carcinomatosis–bearing mice. Adapted with permission from Qi et al. (2020).

Ding et al. reported an NIR afterglow luminescent nanoparticle with aggregation-caused emission (AIE) features (known as AGL AIE dots) (Figure 4A) (Ni et al., 2019). The precursor of phenoxy-dioxetane (mixture 3) and an AIE photosensitizer (TPE-Ph-DCM, λ_*ex*_/λ_*em*_, 465 nm/671 nm) was encapsulated by applying the amphiphilic copolymer lipid—PEG 2000 to generate nanoparticles (AGL AIE dots) (Figure 4B). Ultralong NIR afterglow luminescence imaging achieved low afterglow background noise and ultrahigh tumor-to-liver/spleen signal proportion (34.2/29.1), enabling AGL AIE dots to perform outstandingly in accurate image-guided cancer surgeries in living mice. According to Figure 4C, the microtumors in peritoneal carcinomatosis–bearing mice could be clearly differentiated by NIR afterglow imaging of the AGL AIE dot, but fluorescence imaging (tumor-to-liver/spleen signal ratio, 0.35/0.61) failed to do it. Besides, guided by afterglow imaging, a surgical operation could remove all the tiny tumor nodules. The results indicated that the afterglow luminescent nanoparticle is able to break the main barrier of signal disturbance in the liver for nanoprobes applied in practically and surgically treating the peritoneal metastatic tumor.

To achieve the final objective of accurate image-guided cancer surgery, Ding et al. proposed an alternative category of fluorescent probes with ultrahigh brightness by using the AIE nature and host–guest complexes between calix[5]arene and AIEgen (λ_*ex*_/λ_*em*_, 445 nm/650 nm) for ultrasensitive fluorescence image-guided cancer surgery therapy (Figures 4D,E) (Chen et al., 2020). The restriction of the intramolecular motion of AIEgens contributes to negligible cytotoxic reactive oxygen species production. According to *in vivo* research studies with a peritoneal carcinomatosis–bearing murine model, the supramolecular AIE dot has the capacity to effectively accumulate into and illuminate intraperitoneal tumor nodules, and the SBR value for dot-treated mice has been calculated as 48.5 ± 5.6. Under the guidance of superb NIR fluorescence imaging, the tumor removal surgery was effectively performed (Figure 4F).

Tang et al. reported a type of dragonfly-shaped NIR AIEgen (PTZ-BT-TPA, λ_*ex*_/λ_*em*_, 480 nm/650 nm) with a great absorption coefficient and high fluorescence quantum yield for biomedical imaging and surgical navigation (Figure 4G) (Qi et al., 2020). The assembly of hydrophobic AIEgens is performed in the key, and the amphipathic polymer 1,2-distearoyl-sn-glycero-3-phosphoethanolamine-N-[methoxy-(polyethylene glycol)-2000] (DSPE-PEG_2000_) produces the shell (Figure 4H). According to Figure 4I, while blending the surgeon with the fluorescence signal, his experience excised many tumor nodules with a diameter >1 mm. Nevertheless, the fluorescence signal was observed after the unguided surgery. Then, guided by NIR fluorescence, the surgeon implemented the second surgery to eliminate the remaining tumor, particularly the small tumor nodules which were not harvested fully with lack of guidance. Due to great brightness of AIE NPs, many more tiny nodules (diameter <1 mm) than the un-guided group, were caught by the fluorescence image-guided surgery. Well-overlapping of fluorescence and bioluminescence from the resected nodules was found, verifying the greatly increased surgery precision with the help of NIR fluorescence. To be collective, according to these outcomes, the greatly bright AIE NPs can be a potent probe for accurate image-guided cancer surgeries.

### Osteosarcoma Resection

**FIGURE 5:**
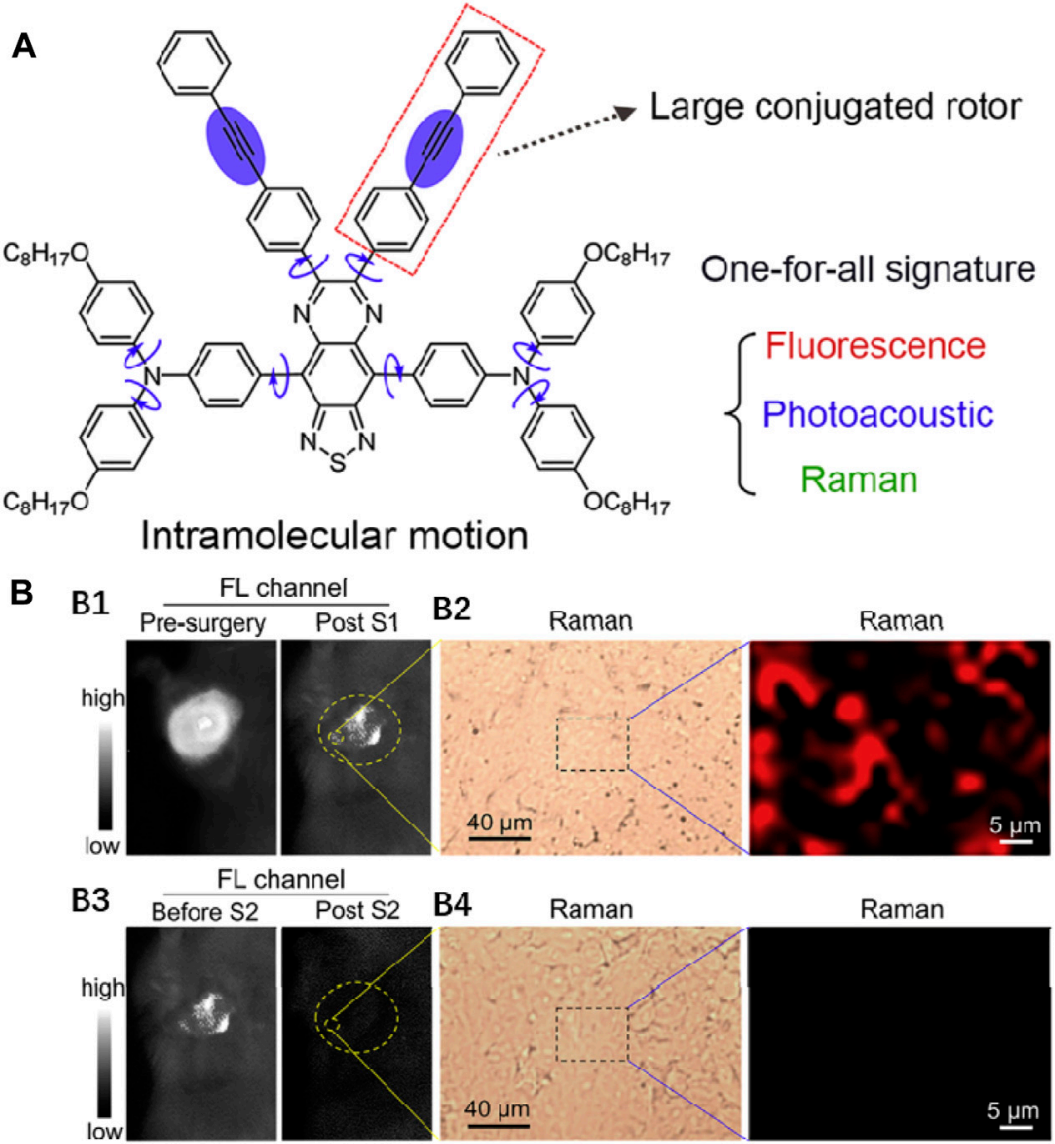
**(A)** Chemical structure of OTPA-TQ3. Adapted with permission from Qi et al. (2019). **(B)** Representative fluorescence images **(B1)** and Raman images **(B2)** of the OTPA-TQ3 NPs-handled tumor-bearing mice before and after the first surgery. Representative fluorescence images **(B3)** and Raman images **(B4)** of the OTPA-TQ3 NPs-handled tumor-bearing mice before and after the second surgery.

Tang et al. disclosed a type of one-for-all organic agent, in which the molecular structure and intramolecular motion can be tuned to synchronously boost the fluorescence, photoacoustic, and Raman nature, for triple modality image-guided accurate cancer surgeries (Figure 5A) (Qi et al., 2019). Utilizing large sensitivity of fluorescence imaging, excellent spatial resolution, and penetration depth of the PA technique, preoperative tumor investigation and the guidance of the surgical scheme was induced by the intravenously administrated OTPA-TQ3 NPs (λ_*ex*_/λ_*em*_, 650 nm/900 nm). In this case, 4T1 tumor–bearing mice were injected into OTPA-TQ3 NPs intravenously for 24 h, followed by NIR fluorescence and Raman imaging, and the vast majority of tumor tissues were removed after performing the first surgery (Figures 5B1B2–6B2). Furthermore, the remaining tiny tumors with diameters of roughly 450 μm after the first surgery treatment could be clearly delineated by intraoperative fluorescence–Raman imaging, and then the residual tumors were completely resected after the second surgery until no fluorescence and Raman signals were observed (Figures 5B3B4–6B4). The image-guided surgery shows that the surgeon can accurately eliminate all the small remaining tumors with quick, real-time, and sensitive fluorescence imaging and high-contrast Raman imaging with zero background and under the help of OTPA-TQ3 NPs, which increase the life span of the mice greatly post-surgery.

**FIGURE 6:**
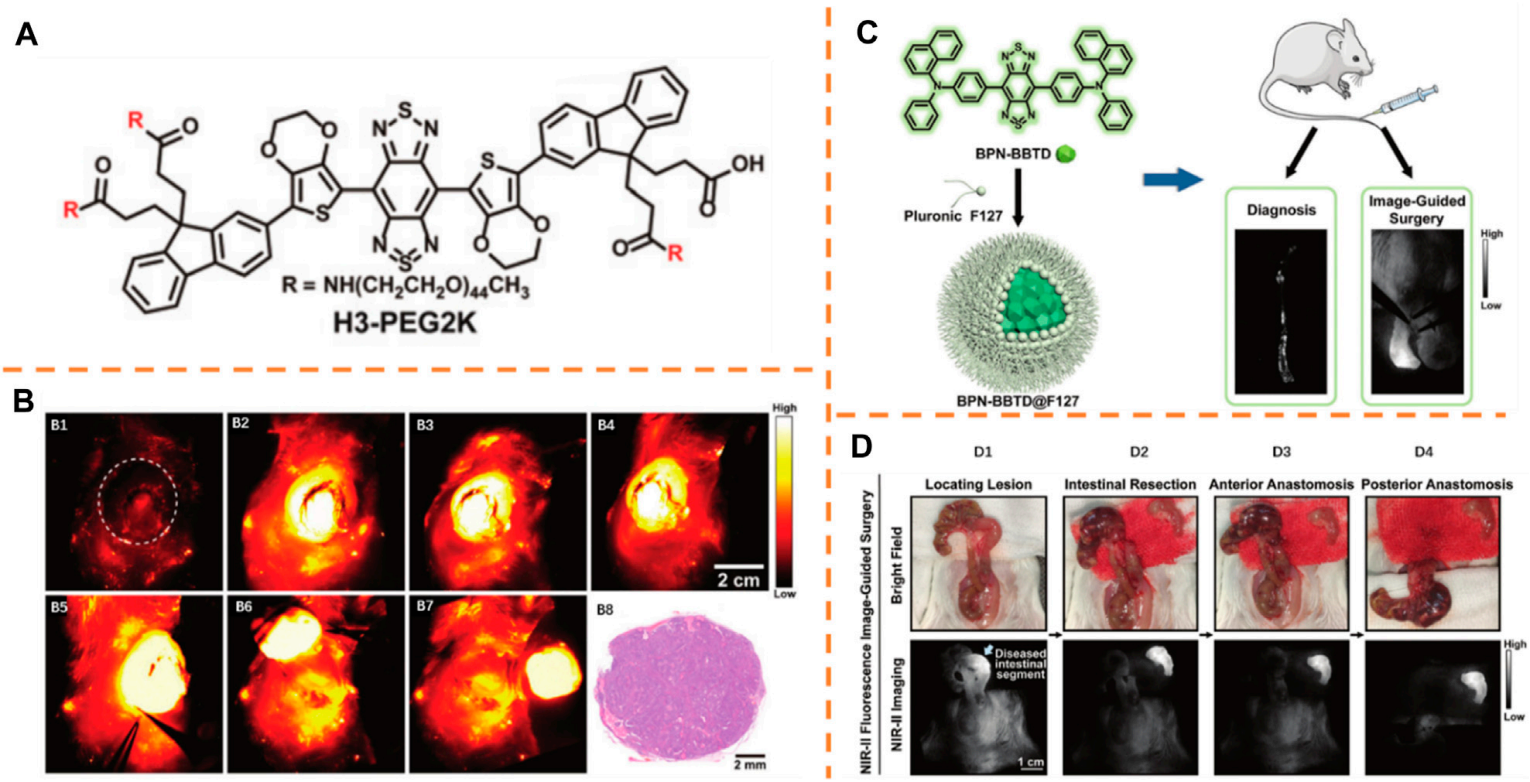
**(A)** Chemical structure of NIR-II probe H3-PEG2k. Adapted with consent from Zeng et al. (2019). **(B) (B1–B4)**: The representative NIR-II fluorescence images. **(B5–B7)**: NIR-II image-guided surgeries after intravenously injecting the probe. **(B8)**: Hematoxylin and eosin (H&E) staining of the carcinoma resected. **(C)** Elementary diagram of the preparation step and usage scenes of BPN-BBTD@F127 NPs. Adapted with consent from Fan et al. (2021b). **(D)** NIR-II fluorescence image-guided resection of bowel parts with serious inflammation.

## NIR-II Fluorescence Imaging Technology for Guided Surgery

Fluorescent imaging of biological systems in the second near-infrared window (NIR-II) can realize micrometer-scale resolution at depths of mm and probe tissue at depths of cm, respectively (Ding et al., 2018b; Cao et al., 2019; Wan et al., 2019; Dai et al., 2021). NIR-II fluorescence image-guided surgical guidance offers one of the most hopeful methods.

### Mammary Carcinoma Resection

Xiao et al. constructed a novel small organic molecule H3-PEG2k (λ_*ex*_/λ_*em*_, 760 nm/1,023 nm) as the biocompatible NIR-II fluorescence imaging agent for *in vivo* imaging and image-guided surgeries (Figure 6A) (Zeng et al., 2019). H3-PEG2k was administrated into a DMBA-caused mammary carcinoma rat by intravenous injection to obtain NIR-II fluorescence images of rat tumors at different time nodes (0, 1.5, 4.5, and 8 h, Figure 6B1–4). The observation of great fluorescence signals was recorded at 1.5, 4.5, and 8 h after injection, and the clear distinction of autonomous tumor description from the nearby normal tissue appeared at 8.5 h (Figure 6B5). The NIR-II fluorescence image-guided surgery was conducted for the complete dissection and removal of carcinoma (Figure 6B5–7). Hematoxylin and eosin (H&E) staining was employed to stain the fluorescent specimen of the overall tumor from the surgery, and there were large amounts of tumor cells found in the overall specimen, but scarce normal tissues were observed in the resection margin of carcinoma (Figure 6B8). This research study offers important guidelines for preclinical research of an NIR-II fluorescent probe as the agent of treating clinical cancers.

### Bowel Resection

Lin et al. constructed a type of aggregation-caused emission (AIE) nanoprobes (BPN-BBTD@F127 nanoparticles, λ_*ex*_/λ_*em*_, 710 nm/930 nm) and first examined the usage value of the nanoparticles (NPs) in supervising the disease progression and the answer to drug violation in inflammatory bowel diseases (IBD) in murine models (Figure 6C) (Fan et al., 2021b). Figure 6D shows the whole course of inflammatory bowel resection. First, strong NIR-II fluorescence was observed in the lesion through the NIR-II fluorescence imaging system. The identification of the colon, partially with great NIR-II fluorescence signals as the seriously diseased colon part (Figure 6D1), was conducted. Second, the aseptic removal of the selective colon part was carried out under the guidance of bright NIR-II fluorescence (Figure 6D2). After resecting the selected colon segment, there were very poor NIR-II fluorescence signals of the stored colon. The anastomosis of the existing intestinal segments with two steeks on the anterior end (Figure 6D3) and the posterior end (Figure 6D4) could obviously be observed. This sensitive and easy approach is greatly underlying with regard to IBD diagnosis and surgical treatment.

### Liver Tumor Resection

**FIGURE 7:**
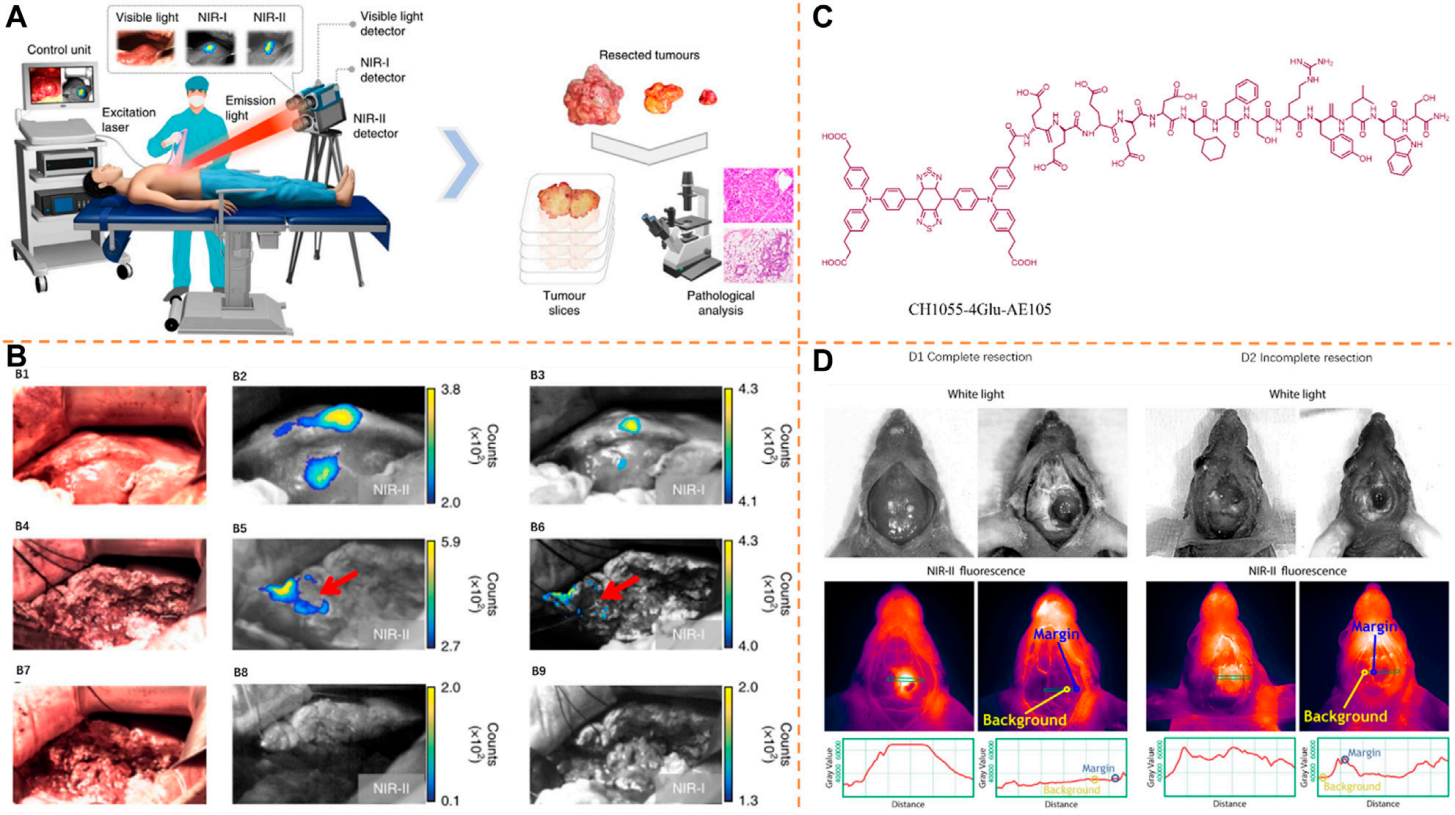
**(A)** In-human liver tumor surgery under the guidance of multispectral optical imaging in the visible and NIR-I/II windows. Adapted with permission from Hu et al. (2020). **(B)** Intraoperative NIR-I/II fluorescence image-guided tumor resection. **(C)** Peptide sequence and the systematic molecular construction of CH1055-4Glu-AE105. Adapted with consent from Kurbegovic et al. (2018). **(D)** For both full resection **(D1)** and inadequate resection **(D2)**, left-hand end images were taken before tumor resection and right-hand end images were taken after resection.

In recent days, Tian et al. (Figure 7A) realized the NIR-II image-guided tumor surgeries in a man (Hu et al., 2020). In their study, FDA-approved ICG (λ_*ex*_/λ_*em*_, 808 nm/980 nm) was adopted as the fluorophore. Integrated detectors in an optical imaging instrument can be employed to simultaneously detect both NIR-I and NIR-II emissions of ICG. Visible light imaging was first received by tumor lesions recognized by surgeons using a multispectral imaging instrument (Figure 7B1). The location of the tumor was identified by NIR-II fluorescence (Figure 7B2). The NIR-I fluorescence allocation mode conformed to that of the NIR-II image (Figure 7B3). Under the guidance of ultrasonography and the visible light image, the tumor was resected and regarded as fully eliminated based on the experience of the surgeons (Figure 7B4). Nevertheless, fluorescence was detected by NIR-II and NIR-I images from the residual tissue (Figure 7B5,6). Then, based on the NIR-I images, the performance of more resection was evaluated. After the second resection, visual investigation showed no remaining tumor (Figure 7B7), and optical imaging displayed no NIR-II or NIR-I fluorescence in the existing tissue either (Figure 7B8,9). With such distinctive advantages, the new NIR-II imaging measures might be highly potential in the improvement of preoperative treatment and intraoperative guidance in the future.

### Glioblastoma Resection

Cheng et al. incorporated NIR-II fluorophore (CH1055) and urokinase plasminogen activator receptor directing peptide (AE105) to develop an oriented NIR-II fluorescent probe (CH1055-4Glu-AE105, λ_*ex*_/λ_*em*_, 750 nm/1,055 nm) (Figure 7C) (Kurbegovic et al., 2018). Guided by NIR-II imaging, the performance of glioblastoma (GBM) resections was evaluated. On bright area images, clear recognition of GBM could not be realized by the midline incision made at the scalp (Figure 7D). By comparison, while imaging with NIR-II, clear visualization of the tumor was obtained with distinct description between the central tumor and the nearby tissue. On the basis of fluorescence imaging, a full resection was regarded due to a margin-to-background proportion (MBR) = 1.0 (margin signal 36,000 Gray and background signal of 35,000 Gray) (Figure 7D1). To be extra, an inadequate resection was conducted with an MBR = 1.5 (residual postoperative fluorescent signal in the periphery/margin with a gray value of 52,500 and a background signal of 36,000 gray, respectively) (Figure 7D2). Under the guidance of NIR-II fluorescence imaging, the orthotopic GBM resections were successfully conducted. As a powerful tool, peptide phage display technology could be used to identify disease-specific antigens that recognize tumor targets. The NIR-II probe and imaging setup have displayed promising preclinical outcomes and translation potential.

### Lymph Node Resection

**FIGURE 8:**
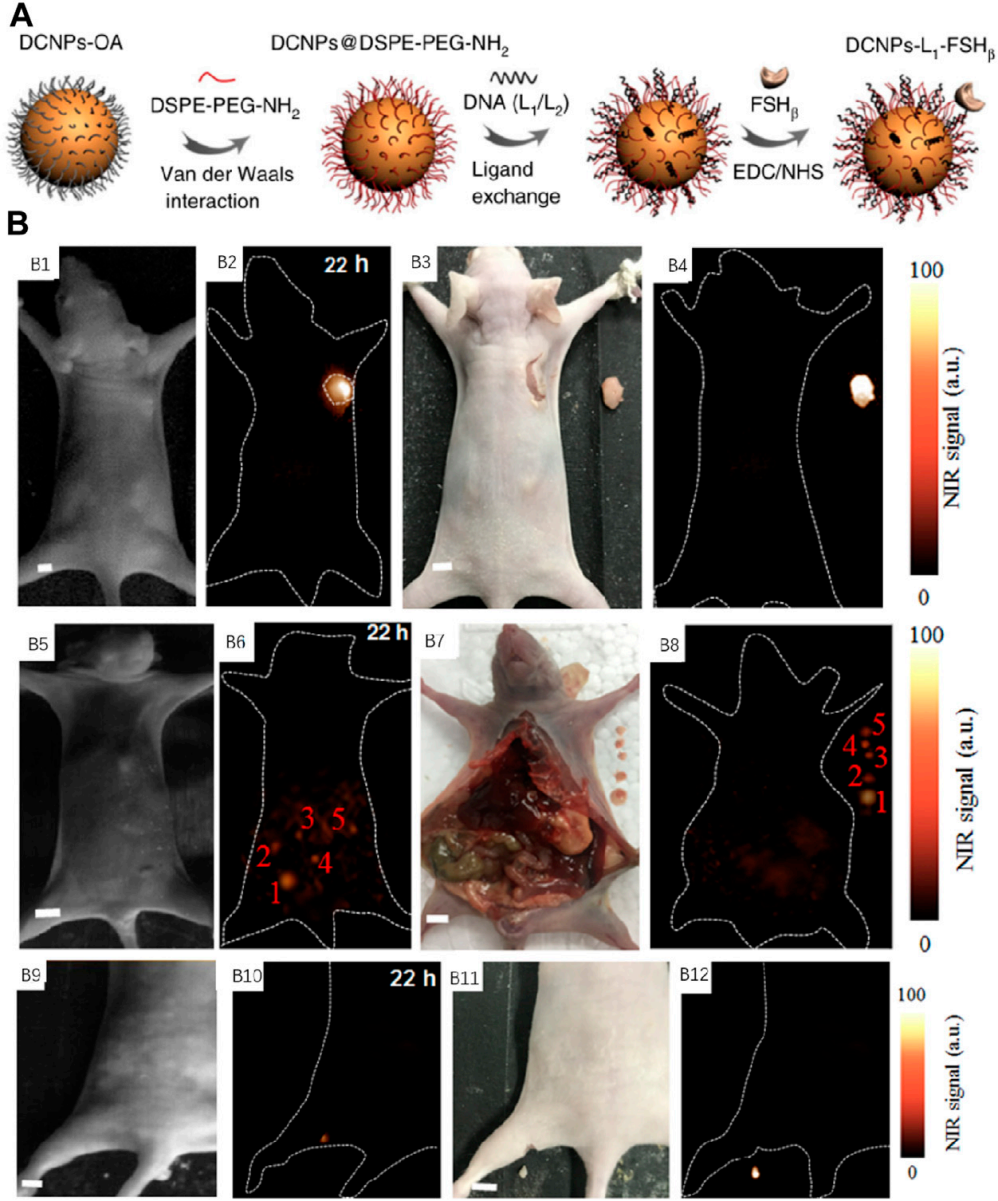
**(A)** Schematic explanation of NIR-II nanoprobe fabrication. **(B)** NIR-II fluorescence image-guided resection. NIR-II fluorescence bioimaging result of the epidermal tumor **(B1,B2)**, digital image **(B3)** and NIR-II fluorescence bioimaging result **(B4)** after surgical resection. The NIR-II fluorescence bioimaging result of peritoneal metastases **(B5,B6)**, digital image **(B7),** and NIR-II fluorescence bioimaging result **(B8)** after surgical resection. The NIR-II bioimaging outcomes of popliteal lymph node metastasis **(B9,B10)**. Digital image **(B11)** and NIR-II fluorescence bioimaging result **(B12)** after surgical resection. Adapted with consent from Wang et al. (2018).

In recent days, Zhang et al. disclosed a new measure by assembling *in vivo* the NIR-II–emitting down shift nanoparticles (DCNPs, λ_*ex*_/λ_*em*_, 808 nm/1,060 nm) functionalized with DNA and directing peptides to enhance the image-guided surgery (Figure 8A) (Wang et al., 2018). Because there is a complementary DNA sequence in these two nanoparticles, efficient tumor retention could be achieved by the assembling. Meanwhile, the quick expulsion of dispersed nanoparticles in the normal tissues from the body by the renal excretory system could be realized because of their small scale, and the non-directed background signal was decreased further. As shown in Figure 8B, DCNPs were successfully applied to map epidermal tumors (Figure 8B1–4), peritoneal metastases (Figure 8B5–8) and popliteal lymph node metastasis (Figure 8B9–12) in intraoperative mice, and fluorescence imaging-guided dissection of the abovementioned tumors in intraoperative mice by injecting DCNPs. By using DCNPs, the precisely preoperation navigation and effectively intra-operation guideline for accurately identifying the tumor margins of the tumors were smoothly actualized *via* NIR-II bioimaging, ensuring complete resection of the tumor.

**FIGURE 9:**
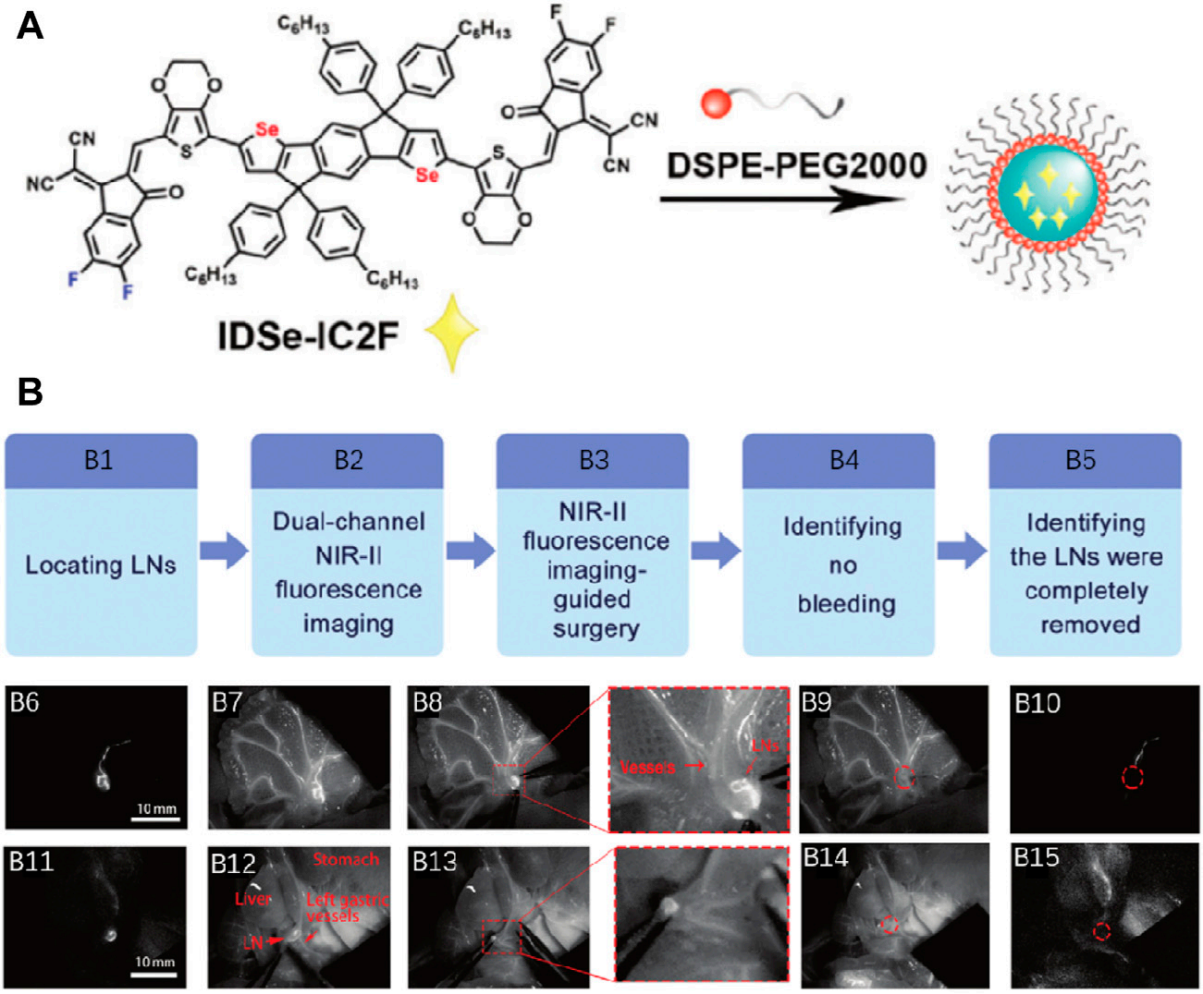
**(A)** Schematic illustration of preparing IDSe-IC2F NPs. **(B)** Double-channel NIR-II fluorescence image-guided lymphadenectomy on rats. Adapted with permission from Fan et al. (2021a).

Lin et al. developed a novel type of organic nanoprobes (IDSe-IC2F nanoparticles, λ_*ex*_/λ_*em*_, 793 nm/1,010 nm) with excellent NIR-II fluorescence and photothermal nature (Figure 9A) (Fan et al., 2021a). For assessing the usage value of double-channel NIR-II fluorescence imaging, Lin et al. conducted the LNs resection directed by this imaging system. Figure 9B1–5 showed major procedures, which could be briefly defined as follows: location identification of LNs, double-channel NIR-II fluorescence image-guided resection, confirmation of no active bleeding, and identification of no remaining LNs. The injection of TQ-BPN NPs and IDSe-IC2F NPs into the tail vein and tiny intestine follicle was conducted. With laser irradiation of 793 nm, mesenteric LNs and the lymphatic vessels could be accurately localized and rapidly found (Figure 9B6). Then, the main blood vessels (Figure 9B7) were detected by turning on the 623-nm LED. Due to the double-channel NIR-II fluorescence imaging, the LNs from the blood vessels could be carefully separated (Figure 9B8). Interestingly, the complete labeling and resection of the two LNs were conducted. Under the LED excitation of 623 nm, whether there was active bleeding after surgery could be easily observed (Figure 9B9). Besides, whether LNs remain in the surgical region after the irradiation of 793 nm was also detected (Figure 9B10). The gastric LNs (Figure 9B11–15) were removed with similar modality. Due to other types of NIR-II fluorescent NPs with various optical characteristics, multi-channel fluorescence image-guided surgery was smoothly performed. The introduction of photothermal ablation of metastatic LNs is made as an excellent complement to the surgery. This platform provided a new angle through which we can perform a more accurate surgery.

### Imaging for Osteosarcoma Resection

**FIGURE 10:**
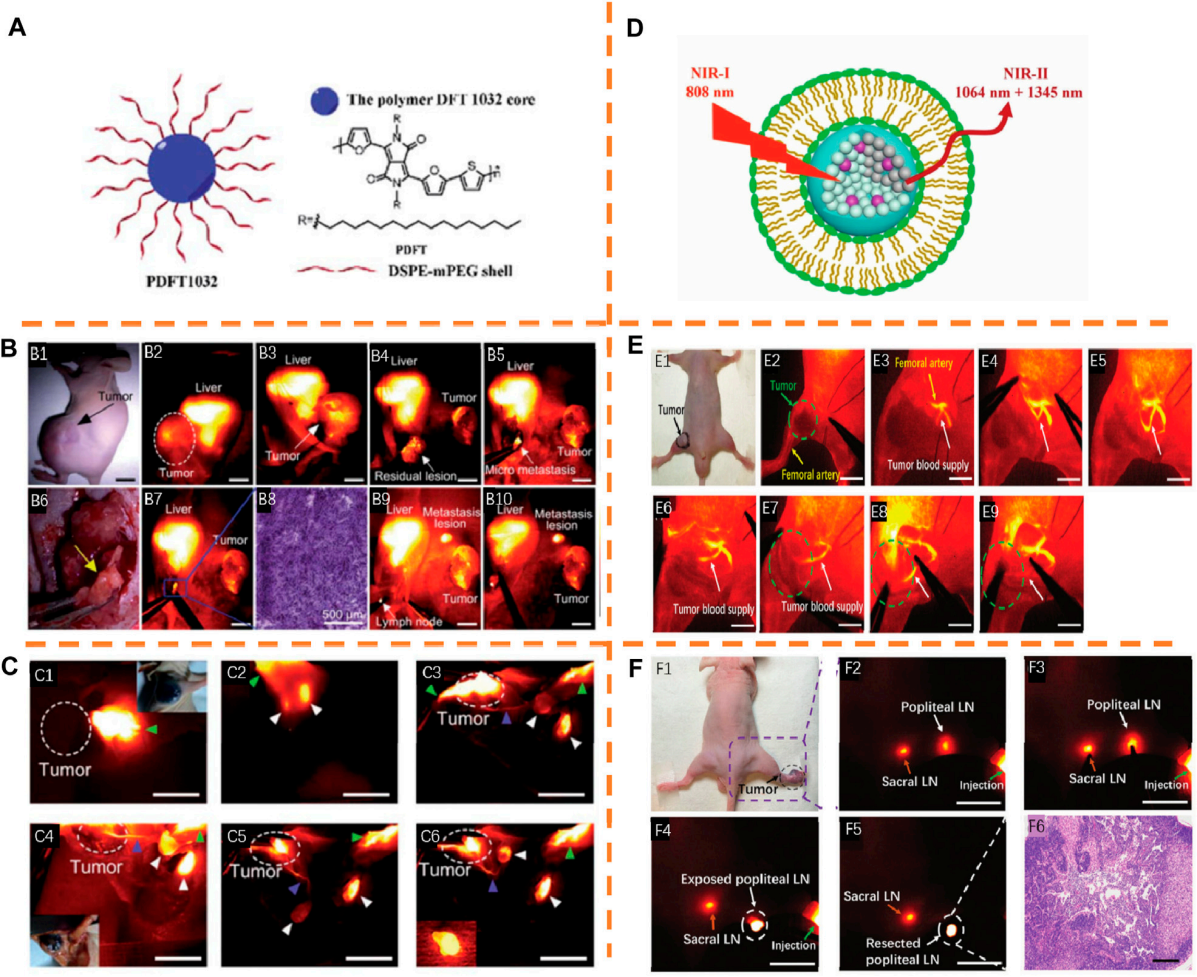
**(A)** Schematic explanation of PDFT1032 fabrication. Adapted with consent from Shou et al. (2018). **(B) B1**: Digital picture of nude mice with an orthotopic osteosarcoma. **B2,B3**: NIR-II images of the whole tumor. **B4**: Resection of the tumor body was performed. **B5**: Mic-NIR-II imaging. **B6**: Bright field photo-metastasis of the tumor. **B7**: NIR-II imaging was adopted to fully resect the micro-metastasis. **B8**: Histological analysis. The visualization and resection of the lymph node were conducted by virtue of PDFT1032 (**B9,B10**). **(C) C1**: Injection of PDFT1032 was administered at the margin of the B16F10 melanoma. **C2**: After 10 min, the clear identification of two axillary lymph nodes was conducted. **C3–C5**: The clear identification of effective lymphatic vessel linking the tumor to the node was performed. The distinct identification of the sentinel lymph node and the secondary lymph node was performed. **C6**: Resection of the sentinel lymph node was performed (inserted image). **(D)** Schematic construction explanation of RENPs@Lips. Adapted with consent from Li et al. (2019). **(E) E1**: Digital picture of nude mice with femur orthotopic osteosarcoma. **E2–E7**: Guided by NIR-II, the main blood supply of osteosarcoma was obviously distinguished from the femoral artery. **E8,E9**: Interim blockage of the main tumor blood vessel was induced by carrying out a vessel clamp, and the signal of blood flow toward the tumor vanished, indicating the smooth realization of embolization surgery of osteosarcoma. **F1**: Digital picture of nude mice with xenograft melanoma. **F2,F3**: Visible popliteal LN and sacral LN at 6 h after injection. **F4,F5**: Resection of popliteal LN. **F6**: histological analysis.

Cheng et al. formulated a diketopyrrolopyrrole-based semiconducting polymer NP (PDFT1032, λ_*ex*_/λ_*em*_, 808 nm/1,032 nm) with a high tumor-to-background ratio (TBR) and smoothly applied it for tumor surgery guidance (Figure 10A) (Shou et al., 2018). The versatile application of PDFT1032 for some significant biomedical uses in the NIR-II window has been shown. For confirming the capacity of PDFT1032 in NIR-II image-guided tumor surgeries, NIR-II imaging, with great comparison (Figure 10B1,2), was employed to visualize the mice with an orthotopic osteosarcoma. Because of the micron-sized spatial resolution and large interim resolution, NIR-II window imaging displayed large sensitivity for the delineation of the orthotopic tumor (Figure 10B3). After completing the image-guided tumor resection, still NIR-II signals remained nearby the knee joint, which were invisible to the surgeon (Figure 10B4–6). Hence, all the residual lesions were eliminated by surveying the resection bed. Interestingly, the identification and complete resection of lesions, such as nearby orthotopic tumor micro-metastasis (Figure 10B5–7) (which was validated through histological exploration further, Figure 10B8) and the lymph node (Figure 10B9,10) were performed. For following up true lymphatic flow and drainage, intradermal injection of PDFT1032 was performed at the tumor margin, and the sharing of the lymphatic path with the tumor (Figure 10C1) would be made. Ten minutes later, the clear identification of the two axillary lymph nodes was realized with the skin intact (Figure 10C2). Guided by NIR-II imaging, the visualization and resection of sentinel lymph nodes were conducted (Figures 10C3–6). PDFT1032 has potential in being extensively applied in clinical imaging and surgically treating malignancy as a highly hopeful NIR-II probe.

Tian et al. reported a kind of excretable NIR-II nanoparticle based on lanthanide, RENPs@Lips (λ_*ex*_/λ_*em*_, 808 nm/1,064 nm) (Figure 10D) (Li et al., 2019). Stimulated by the description of the tumor’s main blood supply, intraoperative guidance for tumor vascular embolization was explored by NIR-II image-guided surgery of femur orthotopic osteosarcoma. The clear discrimination of the main blood supply of the tumor from the femoral artery (Figures 10E2–7) was conducted. Then, the blood flow imitating the clinical vascular embolization step (Figure 10E8) was blocked by implementing a vessel clamp. Remarkably, the signal of the blood flow toward the tumor vanished (Figure 10E9), indicating the smooth realization of embolization surgery for tumor blood supply. Next, a closer sentinel lymph node step in clinical practices was further mimicked under pathological conditions on nude mice with xenograft B16F10 melanoma. Six hours later, intradermal injection of RENPs@Lips was administered at the margin of the melanoma (Figure 10F1), and the sentinel lymph node and secondary lymph node were highly visible (Figure 10F2,3). Guided by NIR-II, the precise dissection of the sentinel lymph node was performed quickly (Figure 10F4,5), and the histological analysis determined that there was melanoma metastasis in the sentinel lymph node (Figure 10F6). RENPs@Lips performs well in the identification of orthotopic tumor vessels intraoperatively and NIR-II image-guided embolization surgery.

**FIGURE 11:**
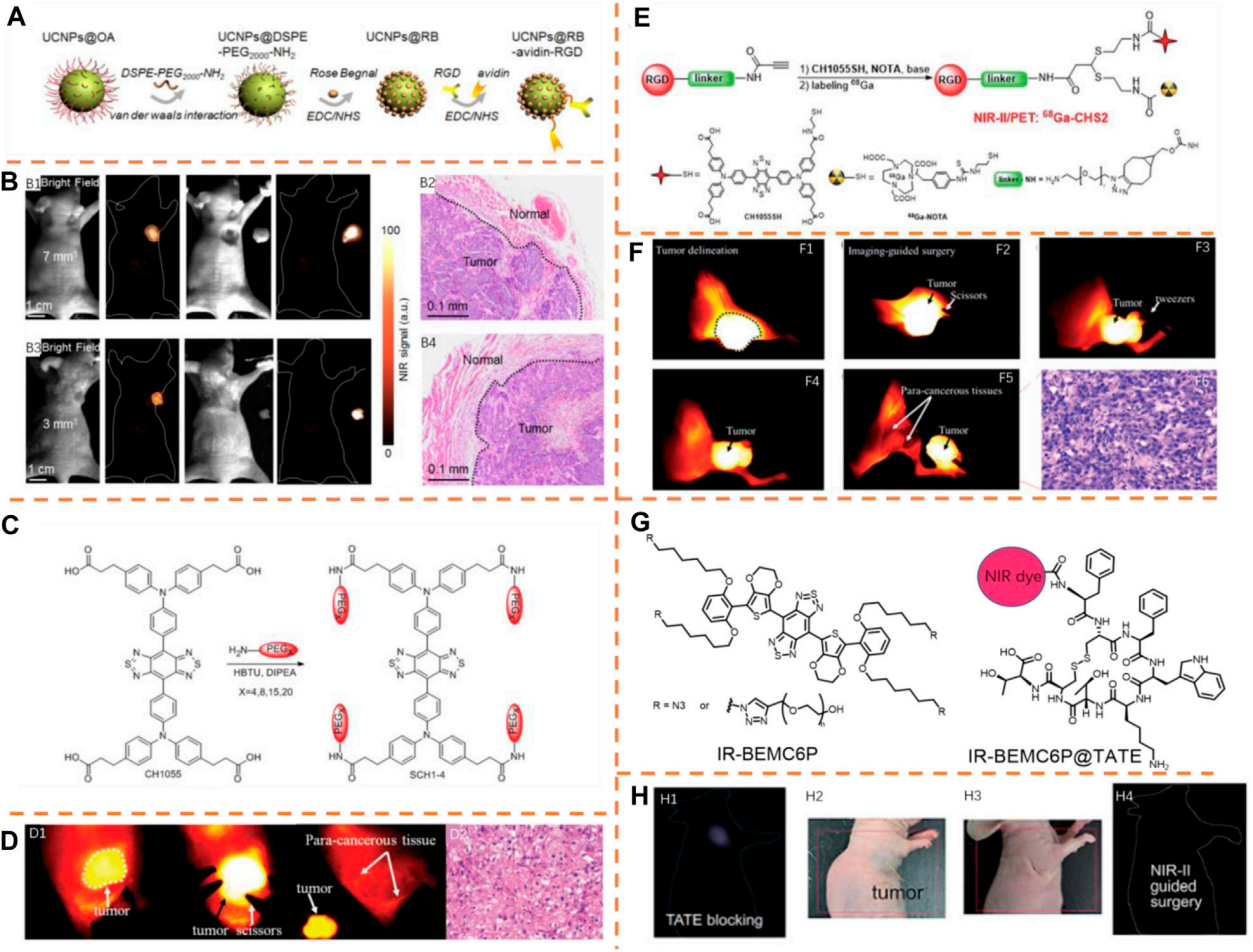
**(A)** Schematic illustration of red blood cell–based probe (RBCp) fabrication. Adapted with consent from Wang P. et al. (2019). **(B)** NIR-II fluorescence bioimaging outcomes (12 h PI) of epidermal tumors with scales of 7 mm^3^ **(B1)** and 3 mm^3^ **(B3)** and NIR-II fluorescence bioimaging outcomes after surgically resecting the tumors. **(B2,B4)** H&E staining outcomes of the tumor and normal tissue border in **B1** and **B3**. **(C)** Integration and self-assembly of NIR-II probes *via* PEGylation: SCH1–SCH4 with scales varying from the nanoparticle to molecule. Adapted with permission from Ding et al., 2018a). **(D) D1**: Image-guided surgery of the HepG2 tumor after intravenously injecting SCH4. **D2**: H&E staining images of the resected tumor tissue. **(E)** Construction of ^68^Ga-CHS2. Adapted with permission from Sun et al. (2018). **(F) (F1–F5)** U87MG tumor description and image-guided surgery at 12 h after the tail vein injection of CHS2. **(F6)** H&E staining outcomes of the tumor. **(G)** Chemical structure of IR-BEMC6P and the conjugate plan of IR-BEMC6P@TATE. Adapted with permission from Liu et al. (2021). **(H) H1**: NIR-II imaging of the IR-BEMC6P@TATE injection after being blocked by the TATE peptide only. Digital picture of the tumor xenograft mouse model before **(H2)** and after **(H3)** NIR-II guided surgeries. **H4**: NIR-II image-guided surgeries for excising the tumor.

Functionalized red blood cells with amine-modified upconversion nanoparticles (UCNPs) were fabricated by Liu et al. as a red blood cell–based multimodal probe (RBCp, λ_*ex*_/λ_*em*_, 808 and 980 nm/835 nm) for NIR-II luminescence-guided accurate tumor resection with a laser irradiation of 808 or 980-nm and laser activated O_2_ production to assist PDT treatment for popliteal lymph node metastasis (Figure 11A) (Wang et al., 2019). NIR-II bioimaging can be employed for the visualization of the full summary of tumors with ∼7 and ∼3 mm^3^ in this superior time window (Figure 11B1,3). Guiding NIR-II fluorescence bioimaging, the investigation into H&E staining was conducted after resecting all tumors, indicating the accurate recognition of tumor margins with various volumes (Figure 11B2,4). In addition, the intraoperative imaging application of the RBCp in a liver popliteal lymph node metastasis model indicates that the metastatic lesion could be precisely identified as well as totally removed by the guidance of NIR-II fluorescence bioimaging in the optimal surgical window. Thus, the RBCp could illustrate accurate identification of solid tumor margins for the guidance and optimization of therapeutic steps.

Sun et al. formulated a unique PEGylation measure and researched the association between the molecular scale and physical and chemical features (Figure 11C) (Ding et al., 2018a). They applied the CH1055 scaffold as the reference for the accurate adjustment of the NIR-II dyes (SCH1–SCH4) from single molecules to nanoparticles, which perform excellent pharmacokinetics and great tumor uptake of organic small molecular probes using the PEGylation strategy. Due to excellent photostability and urine excretion of SCH4 (λ_*ex*_/λ_*em*_, 739 nm/1,050 nm), it was employed for recognizing the liver tumor and guiding the surgery. The clear distinction of tumor delineation from normal tissues was made successfully, and the tumor tissues were completely treated and removed, along with the decrease of the SBR value from 7.2 to 1.1 (Figure 11D1). After this surgery, the surgical tumor tissues were histologically analyzed, which indicated the features of cancer histology (Figure 11D2). Furthermore, SCH4 was also applied to the detection of liver fibrosis distinguished from the surrounding normal tissues in the NIR-II region. Thus, SCH4 can be promising in the research study on animal liver disease models.

The NIR-II/PET probe ^68^Ga-CHS2 (λ_*ex*_/λ_*em*_, 808 nm/1,055 nm) was generated by Sun et al., which could enhance surgical accuracy with a superior SBR value (Figure 11E) (Sun et al., 2018). They formulated a novel dual-mode imaging platform which adds thiols to propylamine stents with base catalysis, realizing various efficient and selectively assembled thiol units with no protective measures. PET imaging and NIR-II FI were conducted on U87Mg-tumor–bearing mice by ^68^Ga-CHS2. Motivated by these hopeful outcomes obtained in *in vivo* PET/NIR-II images, the in-depth evaluation of CHS2 was conducted for follow-up of the accurate description of tumor lesions, resection margins, and image-guided surgeries. The injection of U87MG tumor–bearing mice was administered with 100 mg of CHS2, and the notable identification of tumor description could be realized from the tumor-bearing mice. The SBR value was 4.75 ± 0.22 at 12 h after injection, and while dissecting and removing the tumor from the soft tissue in the leg area, the SBR value declined to 1.16 ± 0.27, showing thorough dissection of the tumor (Figure 11F1–5). After this surgery, surgical tumor tissues were also histologically analyzed, and the features of cancer histology were confirmed (Figure 11F6).

Guided by NIR-II imaging and white light, Chen et al. conducted a tumor excision surgery using an NIR-II fluorophore (IR-BEMC6P, *λ*_ex_/*λ*_em_, 808 nm/1,025 nm) conjugating octreotate (TATE) peptide with outstanding optical and biological properties, such as high quantum yield (1.8%), rapid renal excretion, and minimal hepatic uptake (Figure 11G) (Liu et al., 2021). The AR42J tumor mice injected with a blockage dose of the free IR-BEMC6P without conjugation of TATE showed a very low tumor fluorescence signal, proving the specific tumor-targeting ability of IR-BEMC6P@TATE (Figure 11H1). Besides, a tumor excision surgery was conducted with collaborative NIR-II imaging and white light guidance, which is of advantage to reduce interference and clearly distinct the tumor margin (Figure 11H2–4). The peptide IR-BEMC6P@(RGD, TATE) probes could quickly be excreted renally by comparison with the long-time liver cumulation of representative antibody–dye conjugates. These quickly excreted, greatly secure integrin/somatostatin receptors targeting NIR-II probes promote the clinical interpretation of NIR-II molecular imaging in diagnosing cancer and performing imaging guidance for treatment.

### Imaging for Peritoneal Tumor Resection

**FIGURE 12:**
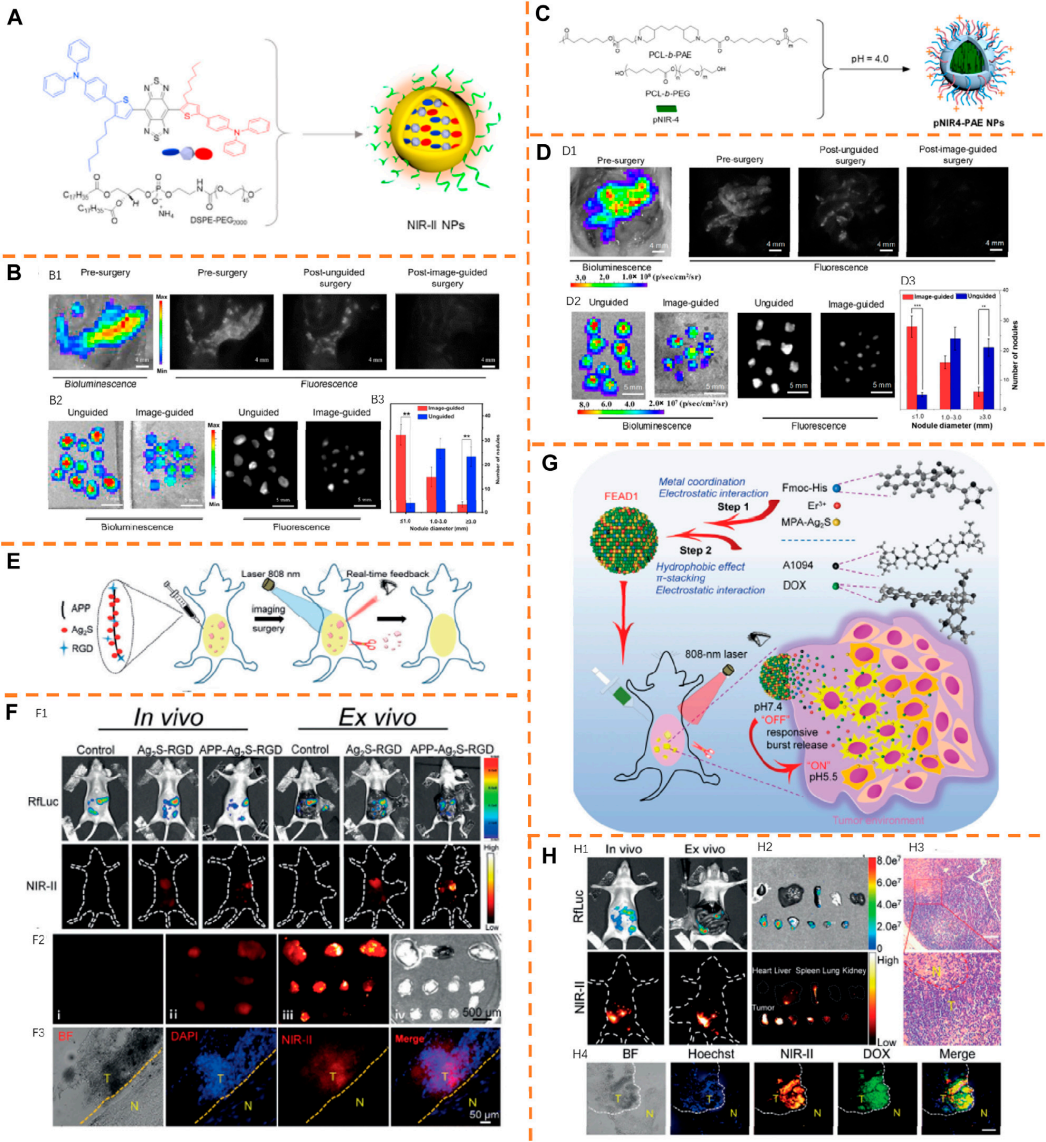
**(A)** Preparation of 2TT-m, oC6B NPs with amphiphilic polymer DSPE-PEG_2000_. Adapted with permission from ref (Liu et al., 2020). **(B) B1**: Bioluminescence and NIR-II imaging of the abdominal cavity before and after resecting the tumor. **B2**: Bioluminescence and NIR-II fluorescence signals of eliminated nodules of unguided and 2TT-m, oC6B NPs–guided groups. **B3**: Histogram of nodule diameters resected from fluorescence-guided and -unguided groups. **(C)** Schematic illustration of fabricating pNIR4-PAE NPs. Adapted with permission from Wen et al. (2019). **(D) D1**: Bioluminescence and NIR-II imaging of the abdominal cavity before and after resecting the tumor. **D2**: Bioluminescence and NIR-II imaging of the resected nodules of unguided and pNIR4-PAE NPs–guided groups. **D3**: Statistical information of the resected nodule diameters. **(E)** Schematic explanation of the NIR-II fluorescent nanochain probe APP-Ag_2_S-RGD for image-guided peritoneal carcinomatosis surgeries. Adapted with permission from Ling et al., 2020. **(F) F1**: Bioluminescence and NIR-II pictures for preoperative and intraoperative phases after IP governance of either Ag_2_S-RGD or APP-Ag_2_S-RGD. **F2**: NIR-II fluorescence images of tumor nodules excised with the naked eye (i) and NIR-II fluorescent imaging guidance (ii: Ag_2_S-RGD, iii: APP-Ag_2_S-RGD); iv) bright-field image associated with the (iii). F3: Bright-field and fluorescence images of the tumor slice procured from the APP-Ag_2_S-RGD-guided surgery using a multi-channel fluorescence microscope. **(G)** Schematic illustration of constructing FEAD1 and ability to accurately diagnose and treat the tumor in peritoneal metastasis. Adapted with consent from Ling et al. (2020). **(H) H1**: NIR-II and bioluminescence imaging of preoperative phasing of intraperitoneally injecting the peritoneal metastasis model of FEAD1. **H2**: NIR-II fluorescent and bioluminescence imaging of tumor nodules excised. **H3**: H&E staining of the tumor slice. **H4**: Fluorescence imaging of the tumor slice procured from the tumor nodules excised.

Tang et al. synthesized the Janus NIR-II molecule 2TT-m, oC6B, further wrapped in the amphiphilic polymer DSPE-PEG_2000_ affording 2TT-m, oC6B nanoparticles (λ_*ex*_/λ_*em*_, 777 nm/1,059 nm) for tumor NIR-II image-guided surgery (Figure 12A) (Liu et al., 2021). For manifesting the feasibility of 2TT-m, oC6B NPs to distinguish the stubborn tumor, the peritoneal carcinomatosis–bearing murine model was used because of the presence of a lot of tumor modules of different scales in the peritoneal cavity. According to Figure 12B1, there was well-overlapping of the fluorescence signal with the bioluminescence signal because of the improved permeability and retention (EPR) role and large brightness of the NIR-II AIEgen NPs. There was a research study conducted on the usage of 2TT-m, oC6B NPs for fluorescence-guided tumor resection. According to Figure 12B1, with no guidance of the fluorescence signal, many relatively large tumor nodules with diameter >1 mm were eliminated by the surgeon. Nevertheless, a lot of dispersive fluorescence signals were observed in the peritoneal cavity after the unguided surgery, showing the residual tumor nodules. Directed by the NIR-II fluorescence signal, a second surgery was conducted, so as to eliminate tiny tumor nodules indistinguishable to surgeons. With large brightness of 2TT-m, oC6B NPs, tumor nodules in fluorescence signal-guided surgery (Figure 12B1) were fully removed by the surgeon, which was determined by the bioluminescence image taken again after the surgery. There was thorough overlapping of bioluminescence and fluorescence signals of the eliminated nodules (Figure 12B2), showing the precision of the surgery. It should be noted that more tiny tumor nodules with diameter <1 mm than that of the unguided group were resected by the surgeon significantly (Figure 12B3), displaying improved surgery precision by NIR-II fluorescence.

Very recently, the same group displayed that NIR-II fluorescence intensity of semiconducting polymers could be effectively improved by structure planarization (Wen et al., 2019). This research study successfully actualized the high-definition vascular visualization in the NIR-II window with a planar and tortuous polymer pNIR-4 (λ_*ex*_/λ_*em*_, 750 nm/1,040 nm) (Figure 12C). Tumor cells both *in vitro* and *in vivo* were employed to efficiently internalize the pNIR4-PAE NPs activated for positive charging in a tumor microenvironment, which is conducive to accurate tumor imaging. After intravenously injecting pNIR4-PAE NPs, the perfect co-localization of NIR-II fluorescence of pNIR4-PAE NPs and the bioluminescence signal of luciferase was observed in the peritoneal cavity (Figure 12D1), showing the precise tumor treatment of pNIR4-PAE NPs. After the second round of surgery with the guidance of NIR-II fluorescence, there was reduced tumor pressure. Verified by the bioluminescence signals of all tumor nodules harvested, the tissue resected was actually a tumor, showing the accuracy of the cancer surgery by virtue of pNIR4-PAE NPs (Figure 12D2). Through the quantification of the diameters of the tumor nodules excised, the excision of a more sub-millimeter tumor has been performed under the guidance of intraoperative imaging (Figure 12D3). While promoting the precise recognition of sub-millimeter tumor nodules, pNIR4-PAE NPs can greatly decrease the risk of *in situ* tumor re-occurrence.

Wang et al. achieved accurate tumor removal in peritoneal metastasis by developing an effective measure and creating a new NIR-II fluorescent nanochain probe, APP-Ag_2_S-RGD (λ_*ex*_/λ_*em*_, 808 nm/1,200 nm) (Figure 12E) (Ling et al., 2020). The NIR-II fluorescence signal was obtained with preoperation and intraoperation conditions for the detection of the directing roles of APP-Ag_2_S-RGD and Ag_2_S-RGD (Figure 12F1). By comparing with the mice handled with Ag_2_S-RGD, the NIR-II fluorescence intensity of the tumor handled with APP-Ag_2_S-RGD was greatly enhanced, and the clear identification of the tumor border was realized. After excising tumors with the guidance of NIR-II fluorescence, quantifying the fluorescence intensity of the tumor nodules showed a super high signal proportion between the tumor and normal tissues with APP-Ag_2_S-RGD over Ag_2_S-RGD (Figure 12F2). Besides, according to fluorescence co-localization images of the tumor provided by the APP-Ag_2_S-RGD-guided surgery, tumor tissues with a great growth in nuclear density show a great NIR-II fluorescence signal, whereas it displayed an inappreciable fluorescence signal among normal tissues (Figure 12F3). With the benefits from its flexible geometry and multivalent direction and its distinct NIR-II fluorescent nature, intraperitoneally injecting APP-Ag_2_S-RGD shows great investigation sensitivity while performing surgery on the tumor.

After that, a tumor microenvironment (TME)–activated NIR-II nanotheranostic system (FEAD1, λ_*ex*_/λ_*em*_, 1,094 nm/1,200 nm) (Figure 12G) was developed by the same group (Ling et al., 2020). The preparation of FEAD1 was conducted by the integration into mercaptopropionic-functionalized Ag_2_S (MPA-Ag_2_S) QDs, DOX, NIR absorber A1094, and peptide Fmoc-His into nanoparticles. After injecting FEAD1 into the mice abdomen, the detection of NIR-II fluorescence signals and bioluminescence signals was performed (Figure 12H1). The fluorescent signal gradually grew over time to become maximum (TBR about 7) at 2 h after injection. Guided by the NIR-II fluorescence, the clear profile and excision of metastatic nodules could be conducted (Figure 12H2). Besides, normative H&E staining was carried out, verifying that the excised nodules were composed of tumor tissues (Figure 12H3). Moreover, fluorescence co-localization images of the tumor from the FEAD1-guided excision displayed that tumor tissues with a great growth in nuclear density produce great fluorescence signals from both NIR-II Ag_2_S QD and DOX, whereas healthy tissues (Figure 12H4) emit negligible fluorescence. FEAD1 has high specificity in lighting up the peritoneal metastasis tumor nodules with large sensitivity.

## Summary and Outlook

In this article, we have emphasized recent prime examples of NIR-I/II fluorescence imaging for surgical navigation and the applications in various surgical resections, including orthotopic osteosarcoma, orthotopic liver tumor, orthotopic breast tumor, renal cell carcinoma, brain tumor, inflammatory bowel disease, peritoneal carcinomatosis, metastatic ovarian cancer, and lymph node. NIR-I/II dyes are capable of offering imaging of deeper tissues, and the activatable approach can achieve higher SBR between diseased tissues and normal tissues. Despite the progress showed above, several ongoing challenges shall be acknowledged and settled. 1) To expand the applications of NIR image-guided surgery from preclinical molecular imaging in experimental animal models to clinical research studies, it is essential to know the complicated systems and pathways in living organisms. Of course, reducing the potential toxicity of imaging reagents and excretion efficiency of imaging reagents by renal and hepatic clearance systems in human physiology is also of great importance to the clinical translation. 2) Moreover, there is a shortage of NIR fluorescent probes with targeting ability that has been recognized by clinical doctors, resulting in the limitation of complete visualization of constructions during image-guided surgery. Thus, it is necessary to develop new fluorescent probes with targeting groups to obtain more accurate image-guided surgery. 3) A comprehensive diagnostic system integrating NIR fluorescence imaging and other in-depth penetration imaging modalities will realize more effective investigation and surgery of cancer or other lesions by comparison with single-mode imaging. For instance, photoacoustic imaging, MRI, and CT will also enhance the imaging depth. Taken together, developing NIR probes with superior performance is of great significance and application value for tackling challenges in surgical navigation applications, and some methodological guidance strategies to improve the performance of NIR probes are summarized as following: 1) Expanding the NIR fluorophore-conjugated structure through plane direction instead of chain direction, which can improve the stability and expand the emission spectra simultaneously. 2) Incorporating water-soluble groups (such as sulfonic- and carboxylic acid moieties) into fluorophore structure or nano-modification of small-molecule organic probes to enhance the structure-inherent cancer targeting ability as well as improve the water solubility. 3) NIR fluorescent probes conjugate with other imaging agents, and dual-modal imaging such as FL/PA, FL/BL, or FL/MR imaging can be achieved at the same time, which can improve the accuracy of biomarker detection and disease diagnosis. 4) Introducing a pro-drug into the NIR probes could enable both real-time imaging-guided delivery and pro-drug activation, enhancing therapeutic outcomes and reducing side effects. In conclusion, it is expected that this review will be effective in creating new NIR I/II fluorescent probes and providing more effective and precise guidance in surgical oncology for excellent surgical results.
